# Effects of nutrients and diet on mitochondrial dysfunction: An opportunity for therapeutic approaches in human disease

**DOI:** 10.1016/j.biopha.2025.118493

**Published:** 2025-08-28

**Authors:** Lisa H. Do, Renata T. Da Costa, Maria E. Solesio

**Affiliations:** Rutgers University, Department of Biology and Center for Computational and Integrative Biology, Camden, NJ 08103, USA

**Keywords:** Mitochondria, Mitochondrial physiology, Nutrients, Diet, Therapeutic approaches

## Abstract

Mitochondria play a crucial role in multiple cellular processes beyond the regulation of bioenergetics. These processes range from apoptosis to intracellular signaling. Accordingly, mitochondrial dysfunction has been broadly described in the etiopathology of multiple human diseases, including cancer, diabetes, and all the main neurodegenerative disorders. Therapeutic interventions aimed at modulating this dysfunction are promising for preventing and/or delaying the development of these pathologies. Recent research has highlighted the potential of dietary interventions to modulate mitochondrial physiology. In this text, we critically review the scientific literature available regarding the effects of different dietary interventions (such as caloric restriction, ketogenic diets, increased omega-3 fatty acid consumption, etc.) on some key components of mitochondrial physiology. Despite the significant advancements in the field that we present in this review, critical gaps remain regarding the molecular mechanisms that underlie the effects of these dietary interventions on mitochondrial physiology, especially under pathological conditions. Future research in this field could underscore these mechanisms, paving the road for the use of dietary interventions against mitochondrial dysfunction as valid pharmacological strategies in human disease.

## 1. Introduction

**Fig. 1.**
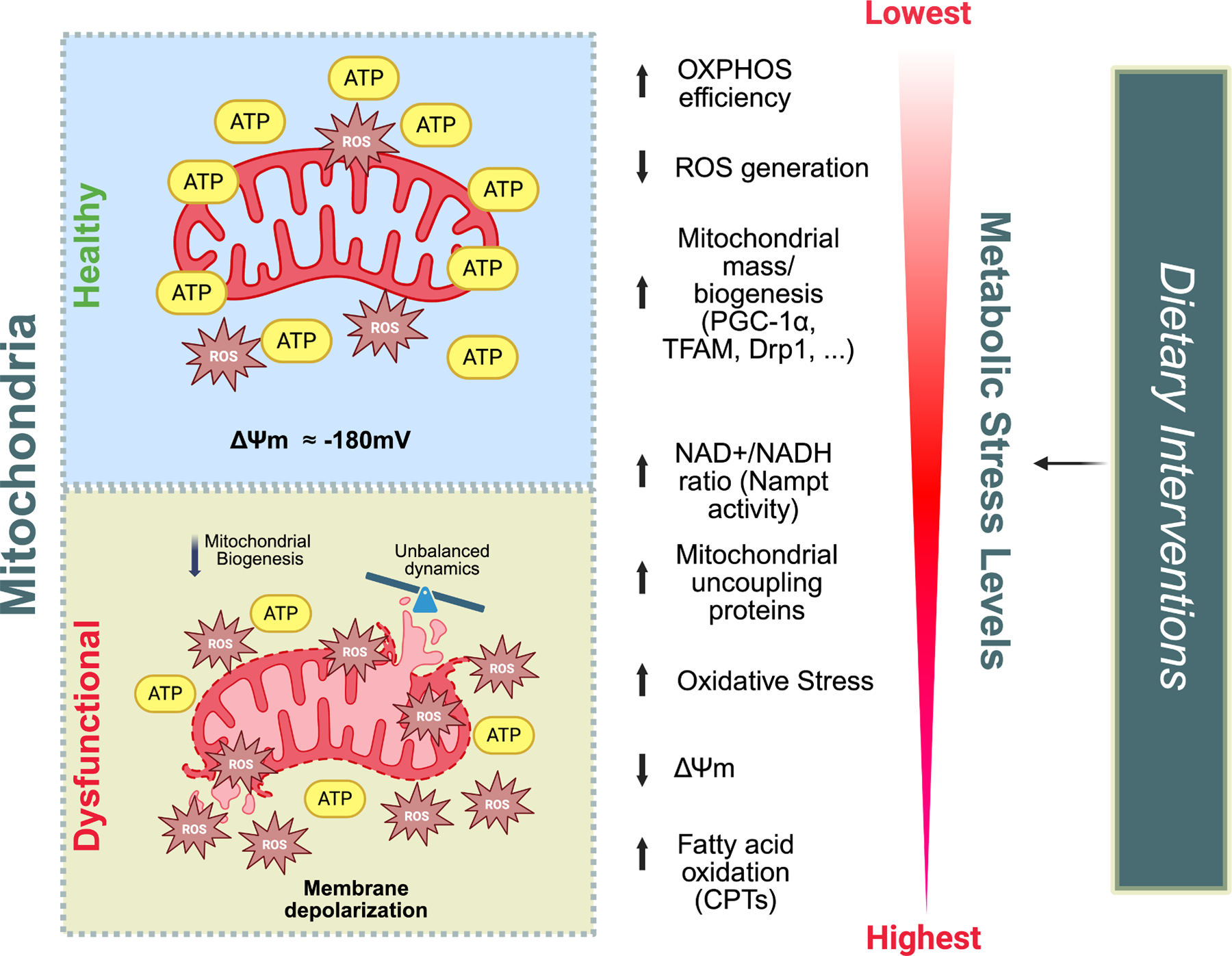
Impact of dietary interventions on mitochondrial physiology. Dietary interventions have various effects on metabolic stress levels; some dietary interventions decrease these levels (for example, CR), while others increase them (for example, high-fat diet). Metabolic stress affects the mammalian cell at different levels, some of which are closely related to mitochondrial dysfunction. The dysfunction of the organelle has been broadly described in the etiopathology of many human diseases. ΔΨm = mitochondrial membrane potential, typically around −180 mV.

Mitochondria are ancient, aerobic organelles that became part of the mammalian cell through an endosymbiotic event approximately 1.5 billion years ago [1]. They are critically involved in many physiological processes in mammals, with bioenergetics being among the most extensively characterized. In fact, the protein components of the electron transport chain (ETC), which is the site of oxidative phosphorylation (OXPHOS), the primary producer of ATP in mammalian cells, are anchored in the inner mitochondrial membrane [2,3]. Beyond bioenergetics regulation, mitochondria are critically involved in many other cellular processes, ranging from the activation and regulation of apoptosis [4] to cell signaling [5,6], and neuronal senescence [6,7]. Mitochondrial involvement in these processes underscores the importance of proper mitochondrial function for maintaining cellular homeostasis [8]. Accordingly, mitochondrial dysfunction has been broadly described in the etiopathology of many human diseases, including cancer [9], diabetes [10], COVID-19 [11], cardiac disorders [12], and all the major neurodegenerative disorders [13–20] (Fig. 1).

In recent decades, multiple research groups have sought for therapeutic approaches to counteract mitochondrial dysfunction, which is a complex and multifaceted process. Two examples include the use of mitochondrial-targeted antioxidants, such as MitoQ [21], in models of human pathologies; and repurposing existing drugs to prevent mitochondrial dysfunction in neurodegenerative disorders [22,23]. The study of the effects of different nutrients and diets on mitochondrial physiology, both under healthy and pathological conditions, has also been investigated. Despite these efforts, critical gaps remain in our understanding. For instance, the long-term effects of dietary interventions on mitochondrial homeostasis and overall mammalian metabolism still remain poorly understood. While various studies have explored short-term dietary impacts, few demonstrate sustained effects on mitochondrial function. Additionally, some studies show how different diets and nutrients influence mitochondrial physiology; however, the molecular mechanisms behind most of these effects still remain poorly understood. Lastly, the presence of some apparently contradictory findings in the literature highlights the complexity of the effects of dietary restrictions on mitochondrial physiology, especially under pathological conditions. For example, while ketogenic diets (KDs) have been shown to improve mitochondrial efficiency and function in neuronal models [24–26], they have also been associated with adverse effects on cardiac mitochondria [27,28], raising concerns about their long-term safety. Similarly, while antioxidants are generally considered beneficial in reducing mitochondrial oxidative stress [20,29–31], some studies suggest that prolonged antioxidant supplementation may suppress mitochondrial biogenesis [32,33], potentially impairing cellular adaptation to metabolic stress. These discrepancies highlight the need for further studies into the intersection between mitochondrial physiology, dietary interventions, and human pathology.

In this bibliographical review, we critically discuss the field of dietary interventions and mitochondrial physiology. A better understanding of their intersection could pave the way for improving our knowledge of the physiology of the organelle, potentially contributing to underscoring new pharmacological strategies to prevent the onset and/or the progression of some of the most common human pathologies.

## 2. Effects of specific diets on mitochondrial function

Dysregulated bioenergetics and increased reactive oxygen species (ROS) generation are often closely interconnected and commonly present in human disease [34]. Therefore, preventing this dysregulation could present therapeutic potential. Interestingly, the effects of specific diets on mitochondrial bioenergetics and the oxidative status of the mammalian cell have already been demonstrated. For example, Bruckbauer and Zemel addressed the impact of high dairy consumption on Sirtuin (Sirt) 1 activation and mitochondrial biogenesis. To do this, they used human muscle and adipose tissue, and an *in* and *ex vivo*/*in vitro* approach. Specifically, overweight and obese subjects were fed a high dairy or a soy-based diet for four weeks. Subsequently, serum samples were collected and used to treat cultured human adipocytes and muscle cells. Their results show that serum from the high dairy diet group significantly increased Sirt1 activity and gene expression in both adipocytes and muscle cells. In the same samples, increased mitochondrial biogenesis, which was shown by the upregulation of key genes such as *PGC-1α*, *UCP2*, *UCP3*, and *NRF1*, was also observed. These findings suggest that dairy diets can be involved in the regulation of Sirt1-mediated mitochondrial function [35].

Another example can be found in the study conducted by Garcia-Roves et al. The authors showed that high-fat diet-fed rats receiving daily heparin injections to increase plasma-free fatty acids exhibited increased expression of enzymes involved in fatty acid oxidation, the citrate cycle, and OXPHOS, along with a significantly increased mitochondrial DNA (mtDNA) copy number [36]. The authors also reported increased activation of the peroxisome proliferator-activated receptor δ (PPARδ), which is often associated with increased mitochondrial biogenesis [37]. Activation of PPARδ leads to a gradual increase in mitochondrial content along with peroxisome proliferator-activated receptor gamma coactivator-1α (PGC-1α, a master regulator of mitochondrial biogenesis and oxidative metabolism) protein expression via a post-transcriptional mechanism rather than by increasing mRNA levels [38].

Furthermore, Leduc-Gaudet et al., showed that young rats that were fed with a high-fat diet for 14 days did not display any major changes in body weight, energy expenditure, and muscle mass. However, this diet significantly enhanced the mitochondrial capacity to oxidize fatty acids in muscular regions of the animals, ultimately affecting mitochondrial respiration [39]. Specifically, both basal and maximal respiration rates were increased in the experimental group of rats, compared to the control animals, when they were treated with lipid substrates (malate + palmitoyl-L-carnitine). In contrast, respiration rates with classical complex I and II substrates, as well as mitochondrial content, remained unchanged. The authors of the same study also demonstrated that some of the skeletal muscular tissue obtained from rats fed a high-fat diet exhibits elevated mitochondrial fission and reduced fusion, compared to control samples. Despite observing elevated mRNA levels of mitochondrial uncoupling proteins (UCP2 and UCP3, which can contribute to reducing ROS generation, and to the regulation of energy metabolism [40]) and mitochondrial lipid transport proteins (CPT1b and CPT2), high-fat diet groups showed no changes in mitochondrial ROS generation and coupling efficiency. This seems to contradict previous research studies that link high-fat diets to increased ROS generation. In fact, uncoupling proteins are able to reduce mitochondrial membrane potential by dissipating the proton gradient, which can decrease ROS generation by limiting excessive electron transport chain activity [41]. The increased expression of lipid transport proteins suggests increased mitochondrial fatty acid oxidation. However, the high-fat diet had no effect on mitochondrial ROS generation and coupling efficiency, which is in contrast with findings from other researchers that link high-fat diets to increased ROS generation [42–45]. The different ages of the animal models used in these studies (young vs old), as well as the differences in the specific composition of lipids of the diets, could explain some of these differences. For example, while young rats were used by Leduc-Gaudet et al., in their study which reported no significant increased mitochondrial ROS generation [39]; Vial et al., and Paglialunga et al., used adult rodents, and they described increased high-fat-associated ROS generation [42,45]. Regarding the specific composition of lipids, the results from different groups suggest that diets with increased saturated fatty acids are more often linked to increased ROS generation; while diets containing more PUFAs seem to attenuate increased ROS generation by modulating mitochondrial membrane properties and uncoupling protein activity. Moreover, the simultaneous upregulation of UCPs and lipid transport protein expression may both enhance fatty acid oxidation and function as a protective mechanism to reduce ROS accumulation [46]. The lack of change in coupling efficiency further suggests that mitochondrial function may be selectively enhanced for lipid catabolism without triggering oxidative stress. This could be potentially due to compensatory mechanisms that offset any unbalanced ROS generation.

The relationship between specific diets, and the oxidative status of mitochondria was further investigated by another group of authors. Ribeiro et al., demonstrated that a high-fat, high-cholesterol diet induces significant alterations in gut microbiota in mice. In the same model, increased short-chain fatty acids (such as propionate and butyrate) metabolism was also found. These changes are associated with increased cell death and oxidative stress in areas of the cortex and the hippo-campus in the brain of the animals, consequently inducing disrupted adult neurogenesis [47]. The authors discussed that the molecular mechanism underlying these effects could involve an unbalanced radical scavenger system, as suggested by the observed decreased Sirt3 expression and adaptive increases in total manganese superoxide dismutase 2 (SOD2) levels. In the same study, increased mitochondrial biogenesis, demonstrated by increased mtDNA copy number and Tfam (mitochondrial transcription factor A) expression, was also observed in the mice fed with the modified diets, compared to the control animals. Ribeiro et al., concluded that while this increase in mitochondrial biogenesis has positive effects on supporting energy production and neurogenesis, it is also associated with increased production of ROS.

Moreover, Gomes et al., explored the effects of berberine (a plant-derived compound known to regulate blood sugar levels and cholesterol) on another model of a high-fat diet in mice. Their study shows that berberine induces a significant reduction in fasting plasma insulin, which indicates improved high-fat diet-induced hyperinsulinemia and enhanced glucose tolerance, despite minimal changes in fasting plasma glucose levels [48]. In the same study, the authors show that berberine mitigates high-fat diet-associated mitochondrial dysfunction; thereby restoring oxygen consumption levels, ATP production, and ETC activity. These effects were exerted via the regulation of the activity of the Sirt1/AMPK axis. Sirt1 promotes the activation of AMPK, which in turn upregulates the expression of key mitochondrial biogenesis genes in both *in vivo* (mice fed with a high-fat diet) and *in vitro* (C2C12 skeletal muscle cells) models. This upregulation ultimately promotes the amelioration of the high-fat diet-induced mitochondrial dysfunction [48].

Some authors have addressed the effects of KDs on mitochondrial physiology. KDs are based on carbohydrate and protein restriction, along with high-fat intake; they have been broadly used in weight loss diets [49]. The goal of these types of diets is to promote the use of fatty acids as the primary source of energy, inducing ketosis in the metabolism of many human tissues [50]. Olive et al., investigated the effects of β-hydroxybutyrate supplementation on mammalian mitochondrial biogenesis and dynamics. To do accomplish, they used a mouse model in which mitochondrial dysfunction resulted from the mutation of an mtDNA repair enzyme (mutUNG1). Their results showed that the ketone body β-hydroxybutyrate induces a significant upregulation of the mitochondrial UCP2 protein, particularly in pyramidal neurons. This increased expression was accompanied by a protective response against oxidative stress [51]. In fact, supplementation with β-hydroxybutyrate not only restored mitochondrial respiration, which was compromised by increased oxidative stress, but also enhanced mitochondrial biogenesis through the upregulation of key regulators such as PGC-1α and Drp1. Lastly, the results show that by improving the NAD+/NADH ratio, β-hydroxybutyrate also promoted mitochondrial function, mitigating oxidative damage. These findings support the strong neuroprotective role of UCP2 that other researchers have already suggested [52].

Using a similar model of KD (β-hydroxybutyrate diet supplementation), Xu et al., studied the effects of this diet on cardiac health and mitochondrial biogenesis. To conduct their experiments, the authors used isolated cardiac fibroblasts and cardiomyocytes from Sprague-Dawley rats as a model. In this case, the animals were fed a KD and intraperitoneally injected with β-hydroxybutyrate. The authors showed that β-hydroxybutyrate offers certain benefits under pathological conditions. However, long-term exposure to elevated levels of β-hydroxybutyrate led to impaired mitochondrial biogenesis, ultimately contributing to serious adverse cardiac outcomes, including fibrosis and increased cardiomyocyte apoptosis. Specifically, the authors demonstrate that deleterious effects on mitochondrial biogenesis are mediated by the activation of Sirt7 as a consequence of increased β-hydroxybutyrate levels [27]. Sirt7 is a protein involved in mitochondrial biogenesis [53]. The activation of Sirt7, via a mechanism also involving the inhibition of the histone deacetylase 2 (HDAC2), downregulates the expression of key mitochondrial proteins, ultimately reducing mitochondrial content and increasing cardiac fibrosis in cardiomyocytes. The authors also described a deleterious effect of elevated β-hydroxybutyrate levels and cardiac fibrosis markers in patients with atrial fibrillation. These last findings raised concerns regarding the potential adverse effects of the use of a long-term KD.

Obesity is closely related to high-fat diet. Accordingly, the effects of obesity on mitochondrial physiology have also been investigated by some researchers. For example, Heinonen et al., examined the relationship between obesity and mitochondrial function, using subcutaneous adipose tissue obtained from human monozygotic twin pairs discordant for body weight. The authors demonstrated that obese twins exhibit downregulated mitochondrial biogenesis and oxidative metabolic pathways, compared to their lean co-twins. Specifically, obesity induced a significant reduction in both nuclear- and mtDNA-encoded transcripts, including those that code for essential components of the ETC. Accordingly, key cellular processes, including fatty acid oxidation, ketogenesis, and the tricarboxylic acid cycle (TCA), are impaired in the obese individuals [54]. The study also reported a correlation between increased adiposity, insulin resistance, and elevated presence of inflammatory markers, therefore suggesting that mitochondrial impairments in subcutaneous adipose tissue precede the onset of clinical metabolic complications. Lastly, the study highlighted the role of epigenetic modifications in regulating mitochondrial activity, noting that key regulators, such as PGC-1α, were found to be downregulated in obese individuals, compared to their lean counterparts [54].

Another example of the effects of obesity on mitochondrial physiology is found in a study conducted by Anderson et al. The authors show that mitochondrial free radical leak is significantly higher in skeletal muscle from obese subjects, compared to healthy individuals [55]. In the same study, they found that a single high-fat meal increased mitochondrial H_2_O_2_ production in lean individuals by more than twofold, relative to pre-high-fat meal levels. This increased production of H_2_O_2_ persisted for up to five days (the duration of the study), while the subjects remained on the high-fat diet. Using rats as models, the authors also confirmed that a high-fat diet is associated with increased insulin resistance in the skeletal muscle. Interestingly, this insulin resistance was reverted by treatment with SS31, which is a mitochondrial-targeted antioxidant peptide, not naturally found in food. SS31 was administered to the animals intraperitoneally [55]. In the same study, but this time using C57BL/6 J transgenic mice overexpressing the mitochondrial human catalase, the authors demonstrate that these animals are able to preserve insulin sensitivity, despite high-fat feeding. The molecular mechanism underlying these effects may involve the maintenance of physiological levels of Akt phosphorylation, thereby enhancing whole-body insulin sensitivity.

Another example of a dietary intervention that has been employed in the context of mitochondrial physiology is caloric restriction (CR). CR is based on the reduction in overall caloric intake, while maintaining adequate nutrient intake to avoid malnutrition [56]. CR has been widely proposed as a method to delay senescence [57], and its effects on cellular metabolism are well documented [6]. At the mitochondrial level, Yang et al., demonstrated that CR enhances resistance to genotoxic stressors, such as etoposide (a topoisomerase type II inhibitor) [58]. The underlying molecular mechanism involves the role of the mitochondrial NAD+ in promoting cell survival under nutrient deprived conditions. NAD+ is essential for both the TCA and OXPHOS. In fact, dysregulated NAD+ metabolism has been reported in numerous human diseases and is considered a major contributor to cell death resulting from genotoxic stress [59],[60]. The authors demonstrated that during genotoxic stress, mitochondria can maintain physiological NAD+ levels even when cytoplasmic and nuclear NAD+ pools are depleted. Notably, following CR, mitochondrial NAD+ levels are significantly elevated through the regulation of the activity of the nicotinamide phosphoribosyltransferase (Nampt), a key enzyme in the NAD+ biosynthesis pathway. Specifically, they demonstrate that Nampt activation is induced by cellular stress and nutrient restriction, leading to elevated mitochondrial NAD+. The authors also demonstrated that the protective effects of Nampt are mediated via the activation of mitochondrial Sirt3 and Sirt4. In conclusion, this study reveals that CR-induced elevations in mitochondrial NAD+ contribute to enhanced cell survival under stress conditions [58].

Moreover, Bevilacqua et al., examined the effects of short- and medium-term CR on mitochondrial physiology of rat skeletal muscle. The authors showed that CR significantly decreases mitochondrial proton leak from early time points. CR also induces a shift towards increased phosphorylation reactions, promoting OXPHOS efficiency. These changes were associated with a drop in H_2_O_2_ production. Furthermore, increased levels of UCP3 were detected at six months (medium-term CR). However, the molecular relationship between UCP3 levels and the observed changes was not directly addressed [61]. Accordingly, the authors were not able to establish a direct causal relationship between the upregulation of UCP3 levels, changes in H_2_O_2_ production, and mitochondrial uncoupling; even if the role of UCP3 on fatty acid metabolism during CR seems now clear [59,62].

In a study conducted in humans, Civitarese et al., addressed the effects of CR (25 % decreased caloric intake, alone or combined with exercise) on mitochondrial function in human muscle from healthy, overweight but non-obese young individuals. Their results show decreased whole body 24-hours energy expenditure, independently of whether exercise was conducted or not. Moreover, in both cases (when exercise was performed and when it was not), significantly increased mitochondrial mass, indicated by elevated mtDNA levels, was present in the skeletal muscle of the individuals. Increased mitochondrial biogenesis, addressed by analyses of gene expression of PPARGC1A and TFAM; as well as reduction in mtDNA damage, was also observed in the same individuals [63]. Surprisingly, the authors also reported that this increased mitochondrial mass did not induce any significant changes in the activity of the enzymes in the TCA, fatty acid oxidation, and ETC. The authors interpret these results to suggest that CR may lead to enhanced mitochondrial efficiency, allowing mitochondria to perform their physiological functions with less energy expenditure, thereby reducing oxidative stress [63]. To further address the effects of CR on mitochondrial biogenesis, Lopez-Lluch et al. showed that cell lines and primary hepatocytes treated with serum collected from rats subjected to long-term CR show a significantly decreased ROS generation. Moreover, the authors found increased mitochondrial mass in liver tissue from rats undergoing CR [64]. They suggest that the effect of CR on ROS generation may be mediated via a mechanism independent of the antioxidant enzymes but associated with decreased mitochondrial membrane potential and reduced oxygen consumption, while maintaining ATP production. This mechanism seems to involve the regulation of a PGC-1α-dependent pathway. Specifically, CR upregulates PGC-1α expression and its downstream targets, Nrf-1 and Nrf-2, which are critical for mitochondrial biogenesis and function [65,66].

Dietary restriction is a type of diet in which specific nutrients are avoided, but which does not necessarily imply a decrease in caloric intake. The effects of such diets on mitochondrial physiology have also been investigated. For example, Berry et al., using *Caenorhabditis elegans*, showed that mitochondrial membrane potential (Δψm) declines with age by day four of adulthood, and observed that dietary restriction, implemented through bacterial deprivation, significantly mitigates this decline. The authors further state that the preservation of Δψm is essential for the observed lifespan extension and improved motility associated with dietary restriction. Notably, their results indicate that the preservation of Δψm under dietary restriction does not rely on the activity of UCP4 (another crucial protein involved in the maintenance of Δψm [67]), but rather requires the functional activity of the mitochondrial adenine nucleotide translocator (ANT), and inhibitory factor 1 (IF1) [68]. Furthermore, the authors show that fatty acid metabolism is critical for maintaining Δψm during dietary restriction. In fact, inhibition of fatty acid oxidation disrupts Δψm preservation and mitigates the dietary restriction-mediated lifespan extension. Similar effects were observed following treatment of the worms with the potent uncoupling drug FCCP [68].

Differences in specific substrate availability are known to sensitize cells to bioenergetics rewiring, thereby affecting mitochondrial physiology [69,70]. Considering the wide range of dietary habits currently observed in humans, and their potential impact on substrate availability, further investigations in this field will be valuable in determining the specific effects of each of these dietary habits on mitochondrial physiology. Modulating these effects holds promising therapeutic potential for the treatment of various human diseases (Table 1).

In fact, recent studies have reported that certain dietary strategies are linked to delayed neuronal aging and a reduced risk of developing age-related neurodegenerative disease, by maintaining mitochondrial health [71]. For example, some studies have highlighted the beneficial effects of specific diets, such as the KD and CR, in slowing the progression of Alzheimer’s disease by regulating mitochondrial quality and function in the nervous system [72,73]. Moreover, dietary interventions have been shown to have beneficial effects in modulating neuroinflammation, improving cognitive performance, and inhibiting the amyloidogenic processing of amyloid beta in Alzheimer’s Disease [74–76]. Mitochondrial dysfunction is involved in all these processes [77],

The study of the effects of dietary interventions extends beyond neurodegenerative disease. In fact, some authors have also explored how these interventions impact the progression of cancer [78]. Interestingly, a third of all cancer cases are attributed, at least partially, to dietary habits [79,80]. This highlights the potential of dietary interventions in reducing the risk of cancer development. Moreover, there is a strong connection between mitochondrial function and the development of malignant phenotypes in cancer biology [81–83]. In this context, one particularly promising aspect of dietary intervention is to enhance mitochondrial fitness, which could have a beneficial impact on halting the formation and progression of many cancer types. Additionally, another promising application of dietary intervention in cancer biology is linked to an enhanced vulnerability of cancer cells to treatments. Accordingly, dietary interventions could increase the efficacy rate of cancer therapies, such as chemotherapy and radiotherapy [73,78].

## 3. Effects of specific nutrient supplementation (or restriction) on mitochondrial function

Vitamins, trace elements, carbohydrates, and other bioactive substances are crucial for maintaining proper mammalian mitochondrial physiology. Accordingly, the effects of dietary supplementation with some of these specific nutrients on mitochondrial function have been investigated. For example, Lalia et al. demonstrated that supplementation with omega-3 polyunsaturated fatty acids (n3-PUFAs) significantly reduces mitochondrial ROS generation through a mechanism that is not well-understood but appears to be independent of mitochondrial oxidative capacity. The authors also demonstrated that n3-PUFAs supplementation enhances mitochondrial protein synthesis in muscle after exercise [84]. Borja-Magno et al., in another notable study, evaluated the effects of omega-3 supplementation (specifically eicosapentaenoic acid and docosahexaenoic acid) on mitochondrial bioenergetics, using peripheral blood mononuclear cells. The study demonstrates significantly improved mitochondrial function, including enhanced OXPHOS efficiency and reduced oxidative stress following omega-3 supplementation, in obese subjects [85]. Altogether, these findings suggest that omega-3 fatty acids play a crucial role in maintaining proper mitochondrial physiology.

Another example of a study addressing the effects of omega-3 fatty acids on mitochondrial physiology is provided by Vaughan et al. These authors investigated the impact of omega-3 fatty acids and conjugated linoleic acid (CLA) on the metabolism of human muscle cells. While omega-3 fatty acids are known to enhance fat metabolism, the role of CLA in this process remains unclear. In the study, human rhabdomyosarcoma cells were treated with omega-3 fatty acids or CLA for 24 or 48 h. The authors showed that treatment with omega-3 fatty acids increases glycolytic capacity and oxygen consumption, indicating enhanced oxidative metabolism and fatty acid oxidation, factors generally associated with improved mitochondrial physiology. Conversely, treatment with CLA reduces both glycolytic and oxidative metabolism, with higher concentrations shifting metabolism from OXPHOS toward glycolysis. Although both treatments increased mitochondrial content, they did not improve mitochondrial function. In fact, omega-3 fatty acids upregulated PGC-1α and glucose transporter type 4 (GLUT4) expression, suggesting increased metabolic activity and glucose uptake [86].

The effects of vitamin and co-factor supplementation on mitochondrial bioenergetics have also been addressed in recent years. For example, by Shen et al., show that supplementation with R-α-lipoic acid, acetyl-L-carnitine, nicotinamide, and biotin improves glucose tolerance and mitochondrial function (including bioenergetic regulation and oxidative metabolism) in diabetic Goto-Kakizaki rats, a well-known model of type 2 diabetes mellitus. These effects were comparable to those observed with the antidiabetic drug pioglitazone, but notably occurred without the associated weight gain [87].

As previously mentioned, mitochondrial biogenesis is closely linked to both the bioenergetic status and the oxidative state of the cell under both physiological and pathological conditions [88]. Accordingly, several studies have already demonstrated the protective effects of specific nutrients and dietary interventions on mitochondrial biogenesis. For example, using differentiated 3T3-L1 cells, Delcourt et al., conducted an elegant study to evaluate the impact of different carbohydrate sources on adipogenesis. Their results show that partially replacing glucose with galactose in the culture medium promotes mitochondrial biogenesis, leading to a greater number of healthy mitochondria and a more stable mitochondrial network by reducing the stimulation of both mitochondrial fusion and fission. This glucose-to-galactose substitution also results in the downregulation of mitochondrial catabolism and the dicarboxylate transporter, suggesting a decrease in metabolic stress [89]. Moreover, Charlot et al., using C57BL/6 J mice fed with a diet containing octanoic acid (a medium chain fatty acid) for six weeks, reported a significant increase in AMPK activation and higher expression of mitochondrial biogenesis markers (including PGC1α and TFAM [90, 91]), in skeletal muscle [92]. The authors concluded that the observed improvements in oxidative capacity in the mice fed with octanoic acid may result from enhanced fatty acid oxidation, and the activation of mitochondrial biogenesis.

Furthermore, Caro et al., addressed the effects of methionine restriction on mitochondrial function, especially on the regulation of oxidative stress and biogenesis. Their results show that methionine restriction significantly decreases mitochondrial ROS generation and the consequent oxidative damage to mtDNA, compared to other amino acids restriction [93]. The authors also highlighted that methionine restriction enhances mitochondrial efficiency, possibly by activating Sirt1, which is a key regulator of cellular energy homeostasis [6,53,94]. More studies in this field were conducted by Perrone et al. The authors found that methionine restriction upregulates genes involved in mitochondrial biogenesis in white adipose tissue from Fischer 344 rats. This upregulation results in increased mtDNA content and enhanced fatty acid oxidation. The authors describe that these effects are likely driven by adrenergic stimulation, which activates multiple metabolic pathways, including some involved in mitochondrial biogenesis and fatty acid oxidation in specific cell types. While no changes in mtDNA content were observed in the liver and skeletal muscle, these tissues showed enhanced aerobic capacity and metabolic shift toward increased fatty acid oxidation [95].

The effects of supplementation with amino acids other than methionine on mitochondrial physiology have also been addressed. For example, Chen et al., studied whether diet supplementation with arginine for four weeks affects mitochondrial biogenesis. To do this, they used weaning piglets as models. Their results show that supplementation with 1 % arginine significantly enhances the activity of the succinate dehydrogenase enzyme, as well as mitochondrial biogenesis via upregulation of PGC-1α and Sirt1 expression, and enhanced mtDNA content. Interestingly, treatment with rotenone inhibited arginine-induced mitochondrial biogenesis. This suggests that the regulation of mitochondrial function is mechanistically involved in the positive effects of arginine supplementation [96]. Furthermore, Liang et al., addressed the effects of leucine supplementation on C2C12 mouse myotubes. The authors show that leucine significantly activates the Sirt1 and AMPK phosphorylation signaling pathways [97], both critical for proper mitochondrial biogenesis [6,94,98]. Specifically, the authors highlight the activation of Sirt1 as a key event in the effects of leucine on mitochondrial biogenesis. This activation has already been shown to influence the acetylation of transcription factors like PGC-1α, which regulates mitochondrial biogenesis [99].

In another study conducted in mice, Davis et al., showed that treatment for seven days with quercetin, a natural polyphenolic flavonoid with well-described anti-oxidant and anti-inflammatory properties [100], increases the expression of key mitochondrial biogenesis markers, including PGC-1α and Sirt1, as well as increased mtDNA content, in both skeletal muscle and brain tissue [101]. These findings suggest that quercetin supports mitochondrial biogenesis and improves mitochondrial function. These conclusions are reinforced by another study, also involving quercetin supplementation. Specifically, Chia-Ling Ho et al. show that pretreatment of SH-SY5Y cells under H_2_O_2_-induced oxidative stress with quercetin restores the expression of critical mitochondrial biogenesis proteins, including Sirt1, PGC-1α, and TFAM, which were otherwise downregulated by oxidative stress [102]. Additionally, this treatment reduces oxidative stress markers and apoptosis by downregulating beta-secretase-1 (BACE1), an enzyme linked to amyloid-beta production [103], while increasing disintegrin and metalloproteinase domain-containing protein 10 (ADAM10) levels, which contributes to reduced amyloid-beta accumulation [104]. Lastly, Barlett et al., in a study conducted on human skeletal muscle, found that p53 activation (via phosphorylation) is enhanced under conditions of reduced carbohydrate availability and physical exercise, which appears to influence mitochondrial biogenesis. The authors describe that p53 activation in this context may be mediated by AMPK signaling [105]. Specifically, low carbohydrate availability was associated with increased expression of Tfam PGC-1α, and COX-IV.

The role of other natural compounds, such as resveratrol (a polyphenolic compound found in various dietary sources), on mitochondrial bioenergetics has also been investigated. For example, Csiszar et al., demonstrate that resveratrol significantly increases mitochondrial mass, expression of ETC components, and mtDNA content, in cultured human coronary arterial endothelial cells. This effect is likely exerted through the upregulation of biogenesis factors, including PGC-1α, Nrf-1, and Tfam [106]. Moreover, the role of Sirt1 in promoting endothelial nitric oxide synthase (eNOS) activation, and thereby increasing nitric oxide bioavailability, was also demonstrated. In fact, when Sirt1 was knocked down or nitric oxide synthesis was inhibited, the beneficial effects of resveratrol were eliminated. *In vivo* studies conducted using mouse models of type 2 diabetes mellitus show that chronic administration of resveratrol restores mitochondrial biogenesis in the aortas of the animals, suggesting a potential protective role in vascular function [106].

Overall, these studies highlight the potential for certain compounds as innovative approaches for preventing and/or delaying mitochondrial dysfunction in human diseases (Table 2). However, additional research is needed to fully understand the molecular mechanisms underlying these effects, as well as the impact of these compounds on different tissues, and in combination with other dietary interventions.

## 4. Effects of genetic models affecting nutrients and mitochondrial function

Genetic models are frequently employed in various fields of biomedical research, including studies on the effects of nutritional interventions on mitochondrial physiology. For example, Waldhart et al. investigated the impact of glucose excess on mitochondrial function using a thioredoxin-interacting protein (TXNIP) knockout mouse model. TXNIP is a crucial regulator of oxidative stress and glucose metabolism in mammals [107]. The authors focused their studies on brown adipose tissue, due to its high mitochondrial content and its essential role in thermogenesis [108]. Their results show a significant reduction in polyunsaturated fatty acids in the mitochondrial membranes of TXNIP knockout mice [109]. This reduction deleterious impacts mitochondrial integrity and ETC efficiency, leading to decreased heat production, especially under stress conditions, such as cold exposure. Additionally, the TXNIP knockout mice exhibited elevated glucose levels and impaired mitochondrial biogenesis, as evidenced by a reduction in the expression of PGC-1α. The same study shows that a ketogenic diet can partially rescue metabolic dysfunction by increasing the polyunsaturated fatty acids in the mitochondrial membranes, ultimately improving mitochondrial function and promoting a shift from glycolysis towards fatty acid oxidation. This metabolic shift alleviates some excess glucose-mediated metabolic stress, and it enhances mitochondrial biogenesis [109].

To better understand the molecular mechanism that links mitochondrial biogenesis and diet, using *Drosophila* as their model, Baltzer et al. demonstrated that Delg, the *Drosophila* homolog of mammalian Nrf-2α, is crucial for coordinating mitochondrial functions under varying nutrient availability conditions [110,111]. Specifically, the authors show that Delg mutants exhibit reduced mitochondrial number, size, and glutamine metabolism, similar to low-yeast food conditions. The authors interpret these findings as an indication that Delg functions as a transcription factor to regulate mitochondrial activity in response to changes in nutrient levels. Interestingly, despite reduced expression of OXPHOS genes, Delg mutants maintain physiological OXPHOS activity, suggesting that individual mitochondria in these mutants are more active. They also show that Delg is required for Cyclin D/Cdk4-induced mitochondrial biogenesis, linking Delg to cellular growth pathways. Additionally, Delg operates in parallel with Spargel (PGC-1 homolog) in controlling mitochondrial mass and function. These results highlight the novel role of Delg in mitochondrial regulation and its evolutionary divergence from mammalian Nrf-2α mechanisms [110].

## 5. Conclusions

Adequate mitochondrial function is crucial for the maintenance of mammalian metabolism. In fact, mitochondrial dysfunction has been demonstrated as a key contributor to the etiopathology of numerous diseases, including cancer, diabetes, and all the major neurodegenerative disorders [9–11,13–20]. In recent decades, researchers have addressed the potential positive effects of dietary interventions on mitochondrial physiology, using a wide variety of models (Table 3). Moreover, the effects of specific diets on mitochondrial parameters which are dysregulated in specific diseases have also been studied. For example, low-glycemic diets have shown positive effects in diabetic and pre-diabetic patients, via reducing glycated hemoglobin, fasting glucose, body mass index, and cholesterol; even if they show no effect on other important parameters, including fasting insulin, and triglyceride levels [112]. Moreover, the Mediterranean diet, which is rich in antioxidants and monounsaturated fats, has been linked to neuroprotective effects, which seem to be linked to mitochondrial physiology, at least partially [113].

While the efforts to address the effects of different dietary interventions on mitochondrial physiology have been fruitful, further investigation needs to be conducted to better determine the long-term effects of these interventions on the physiology of the organelle. Moreover, a more comprehensive, deeper, and mechanistic understanding of the interactions between mitochondrial physiology and dietary restrictions will be essential for the development of targeted, evidence-based dietary strategies to optimize mitochondrial health and prevent the onset and/or the progression of mitochondrial dysfunction in human pathologies.

## Figures and Tables

**Table 1:** Effects of different dietary interventions on mitochondrial physiology. The effects of the three main dietary interventions that are described in this review (that is, caloric restriction, ketogenic diet, and high-fat diet) on some of the main mitochondrial parameters are summarized in this table. Bibliographical references to the studies where those specific effects were addressed are also included.

Mitochondrial parameter	Caloric restriction	Ketogenic diet	High-fat diet
*Bioenergetics*	Maintenance of NAD+ levels under stress conditions [58].	Improved efficiency in neuronal models [24–26].	Increased expression of enzymes involved in OXPHOS [36].
	Decreased proton leak, promoted OXPHOS efficiency [61].	Restored mitochondrial respiration in models of mitochondrial dysfunction [51].	Increased basal and maximal respiration rates [39].
	Decreased whole body24-hours energy expenditure [63].		
	Increased OXPHOS efficiency without affecting the activity of TCA and ETC enzymes [63].		
*ROS generation/oxidative stress*	Reduced H2O2 generation [61].	Protection against oxidative stress [51].	No changes in ROS generation [39].
	Reduced mtDNA damage, and oxidative stress [63].	Mitigated oxidative damage [52].	Increased ROS generation [42–45]. Increased
	Decreased ROS generation [64].		oxidative stress when high-fat is associated with high-cholesterol [47].
*Mitochondrial biogenesis*	Increased mitochondrial mass [63,64].	Enhanced mitochondrial biogenesis in neurons [51].	Increased mtDNA copy number [36].
			Increased mitochondrial biogenesis [47].
	Increased biogenesis [63].		
		Impaired mitochondrial biogenesis after long-term exposure in cardiac cells [27].	
*Mitochondrial dynamics*	Not addressed in the presented studies.	Upregulated Drp1 in neurons [52].	Elevated fission, and decreased fusion [39].
*Mitochondrial membrane potential*	Increased levels of UCP3 [61].	Upregulated UCP2 [51, 52].	No changes in coupling efficiency, despite increased mRNA levels of UCP2 and UCP3 [39].
			Changes in coupling efficiency [42–45].
*Overall impact*	Protective under stress conditions [58].	Associated to adverse effects on cardiac mitochondria [27, 28].	The different effects might be related to the different ages of the animals and singularities of the high-fat diet.
		Promoted mitochondrial function [52].	
		The effects seem to depend on the specific tissue and the duration of the diet.	

**Table 2:** Effects of nutrient supplementation or restriction on mitochondrial function. The effects of supplementation or restriction of specific nutrients on mitochondrial physiology that are described in this review are also included in this table. Bibliographical references to the studies where those specific effects were addressed are also included.

Interventions	Key Mitochondrial Effects	Molecular Pathways	References
Omega–3 fatty acids (n3-PUFAs) supplementation.	Decreased ROS generation, and increased mitochondrial protein synthesis in muscle postexercise.	Unknown, independent of oxidative capacity.	[85]
Omega–3 supplementation.	Enhanced OXPHOS efficiency, and decreased oxidative stress.	Not specified.	[85]
Omega–3 vs. CLA supplementation.	Omega–3: increased glycolytic capacity, and oxygen consumption. CLA: decreased glycolytic capacity, and oxidative metabolism. Higher concentrations shift metabolism from OXPHOS towards glycolysis.	Omega–3: Upregulated PGC–1α, and GLUT4 expression.	[86]
R-α-lipoic acid, acetyl-L-carnitine, nicotinamide, and biotin.	Improved glucose tolerance, and mitochondrial function.	Not specified.	[87]
Partial replacement of glucose with galactose.	Increased mitochondrial biogenesis, creating a stable mitochondrial network by decreasing both fission and fusion. Downregulated mitochondrial catabolism.	Adipogenesis modulation.	[89]
Diet containing octanoic acid.	Improved oxidative capacity, including increased AMPK activation, and higher expression of PGC–1α and TFAM in skeletal muscle.	Enhanced fatty acid oxidation.	[92]
Methionine restriction.	Decreased ROS generation, and consequent damage to mtDNA. Increased mitochondrial efficiency.	Possibly by activating Sirt1.	[93]
	Upregulated genes involved in mitochondrial biogenesis in white adipose tissue in rats, resulting in increased mtDNA content and enhanced fatty acid oxidation.	Likely driven by adrenergic stimulation.	[95]
Arginine supplementation.	Enhanced activity of the succinate dehydrogenase enzyme, and mitochondrial biogenesis.	Upregulation of PGC–1α and Sirt1 expression, and enhanced mtDNA content.	[96]
Leucine supplementation.	Enhanced mitochondrial biogenesis.	Activation of Sirt1 and AMPK phosphorylation pathways.	[97]
Quercetin supplementation.	Increased the expression of key biogenesis markers, and mtDNA content in both skeletal and brain tissue.	Not specified.	[101]
	Restored oxidative stress-induced decreased expression of critical mitochondrial biogenesis proteins. Downregulated BACE1, and increased disintegrin and ADAM10.	Not specified.	[102]
Reduced carbohydrate availability.	Increased mitochodnrial bioegenesis.	Activation (via pshophorylation) of p53.	[105]
Resveratrol supplementation.	Increased mitochondrial mass, and mtDNA content, upregulated expression of ETC components, and increased mitochondrial biogenesis in the aorta.	Sirt–1 and nitric oxid-dependent.	[106]

**Table 3:** Studies about the effects of different dietary interventions on mitochondrial physiology have been conducted in a wide range of models. This table summarizes the literature that shows the effects of different dietary interventions in the main models used by researchers. Bibliographical references to the studies where each specific combination of dietary intervention and model was used are also included.

Experimental model	Dietary intervention	References
*C. elegans*	Dietary restriction.	[68]
*Mice*	High-fat, high cholesterol diet.	[47]
	Berberine supplementation, high-fat diet.	[48]
	β-hydroxybutyrate supplementation.	[51]
	High-fat diet in a transgenic mice model overexpressing the mitochondrial human catalase,	[55]
	Octanoic acid supplementation.	[92]
	Leucine supplementation.	[97]
	Quercetin supplementation.	[101]
	Resveratrol supplementation.	[106]
	Excess glucose in a thioredoxin-interacting protein knockout mice model.	[109]
*Rats*	High-fat diet.	[36]
	High-fat diet	[39]
	High-fat diet.	[55]
	CR.	[64]
	Diabetes.	[87]
	Methionine restriction.	[95]
*Weaning piglets*	Arginine supplementation.	[96]
*Cells*	Berberine supplementation. Model: C2C12 skeletal muscle cells.	[48]
	Supplementation with serum from rats under CR. Model: cell lines and primary hepatocytes.	[64]
	Omega–3 supplementation. Model: peripheral blood mononuclear cells.	[85]
	Omega–3 fatty acid or conjugated linoleic acid treatment. Model: rhabdomyosarcoma cells.	[86]
	Partial replacement of glucose with galactose. Model: differentiated 3T3-L1 cells.	[89]
	Quercetin supplementation. Model: SH-SY5Y cells.	[102]
	Resveratrol supplementation. Mode: cultured coronary arterial endothelial cells.	[106]
*Human samples*	High dairy, soy-based diet	[35]
	Obesity.	[54]
	Obesity.	[55]
	CR.	[63]
	Reduced carbohydrate availability and physical exercise.	[105]

## Data Availability

No data was used for the research described in the article.
